# A Systematic Review of the Efficacy of Compression Wraps as an Anxiolytic in Domesticated Dogs

**DOI:** 10.3390/ani14233445

**Published:** 2024-11-28

**Authors:** Savannah Mathis, Suzie Schoolfield, Peggy Gross, Margaret Gruen, David C. Dorman

**Affiliations:** 1 Byram Hills High School, Armonk, NY 10504, USA; mathiss25@byramhills.net; 2 College of Veterinary Medicine, North Carolina State University, Raleigh, NC 27606, USA; smschool@ncsu.edu (S.S.); mkgross2@ncsu.edu (P.G.); margaret_gruen@ncsu.edu (M.G.)

**Keywords:** canine anxiety, pressure wraps, anxiolytic, welfare

## Abstract

Many domesticated dogs show signs of anxiety, negatively impacting their own and their owner’s mental and physical health. A systematic review evaluating whether pressure wraps decrease clinical signs of anxiety in dogs was completed to identify relevant studies. The main outcomes of interest included behavioral and physiological measures. A total of 229 studies were identified, of which four met the inclusion criteria. Commercially available pressure wraps and a telemetry vest were used in the reviewed studies. Three experimental studies included brief exposure to recorded firecracker or thunderstorm sounds or separation from their owner as anxiety-invoking stimuli. A non-experimental study evaluated the use of pressure wraps for dogs with thunderstorm phobia living in their home environment exposed to naturally occurring thunderstorms. The risk of bias in domains related to the blinding of investigators or study participants was generally high. Domains with moderate risk of bias often reflected incomplete reporting of research methods and imprecision due to small sample sizes. Most studies reported minimal benefits on behavior or heart rate in the absence of adverse side effects. Our review suggests there is weak and limited evidence to support the beneficial effect of pressure wraps in reducing physiological or behavioral signs of anxiety. Confidence in the available literature is low due to the moderate-to-high risks of bias and inconsistent findings. Further studies are needed regarding the efficacy of these products.

## Simple Summary

Dogs commonly develop anxiety disorders including increased reactivity toward loud noises associated with fireworks, thunderstorms, and other sound sources. Other common anxiety conditions include generalized anxiety disorder and separation anxiety. These anxious episodes can be debilitating with negative impacts on the welfare of the animal and increases in owner stress. A variety of approaches have been considered to reduce canine anxiety, including behavioral modification, the use of anti-anxiety medications, and other alternatives. One such alternative is to use pressure wraps to reduce anxiety. This project systematically reviewed the available veterinary literature to evaluate whether existing evidence supports the use of pressure wraps to reduce anxiety in dogs. We found four published studies examining the use of pressure wraps in different anxiety syndromes. The types of pressure wraps varied among the evaluated studies. Our review indicates that the use of pressure wraps is not associated with adverse side effects. Our review also found limited evidence to support the benefit of using pressure wraps in reducing anxiety in dogs. Further studies are needed to make more reliable and accurate conclusions regarding these products.

## 1. Introduction

Canine anxiety disorders, fears, and phobias are common behavior problems seen in veterinary clinics and hospitals across the United States [1]. These problems include generalized anxiety, separation anxiety, and phobias of specific stimuli such as storms, fireworks, or other noises [2]. A recent survey of Finnish dog owners revealed that over 70% of all dogs had one or more anxiety disorders, with noise sensitivity being the most common anxiety-related trait with a prevalence of 32% in 13,700 Finnish pet dogs [3]. This study used an owner-reported survey to examine seven anxiety-like traits and problematic behaviors. Veterinarian-based diagnoses were not available to confirm these survey results. Anxiety disorders are an important animal welfare concern that diminish the quality of life of domesticated dogs and represent a significant risk factor for the relinquishment of dogs to animal shelters [4]. The development of pharmacological and non-pharmacological interventions could therefore dramatically improve the welfare of affected dogs and their owners.

Treatments for noise-associated disorders and other canine anxiety disorders may incorporate environment management, medication, behavior modification programs, and alternative techniques [5]. Pharmacological approaches have included the use of clomipramine [6,7], trazodone [2], imepitoin [8,9,10], and dexmedetomidine [11,12,13]. Pheromones have also been used to manage noise phobias in dogs with mixed success [14,15,16,17,18].

Moderate-to-deep pressure is an alternative treatment method that has been used in animals to reduce tension and anxiety [19,20,21]. Deep pressure touch is carried by the dorsal column system and may influence reticular formation activity with direct effects on autonomic activity [22]. Deep pressure touch increases vagal tone by influencing both parasympathetic and sympathetic activity, resulting in reduced activation of the stress response [23]. Pressure can be applied in a variety of ways, including the use of weighted materials, capes, or wraps. Several studies have examined the use of garments in dogs in reducing aggression [21], responses to thunderstorm noise [24,25] and firework sounds [26], or anxiety disorders [27]. Our laboratory has shown that dogs wearing telemetry vests had reduced anxiety when presented with recorded thunderstorm sounds [28].

The aim of this present systematic review was to determine whether there was a beneficial effect of a pressure wrap on either physiological measures (e.g., heart rate) or behavior in domesticated dogs presented with an anxiety-inducing stimulus.

## 2. Materials and Methods

### 2.1. Problem Formulation and Protocol Development

The systematic review study protocol was developed using guidelines provided by the Cochrane Collaboration [29]. The protocol detailed the research question and outcomes of interest, outlined a search strategy and the process of data extraction, and provided criteria for rating the quality of evidence (Supplemental Materials). The specific review question and population, intervention, comparator, and outcome (PICO) statement for the systematic review were as follows.

#### 2.1.1. Review Question

Does mild-to-moderate pressure reduce either behavioral or physiological markers of anxiety in dogs with either pre-existing anxiety disorder or those exposed to an anxiety-invoking stimulus?

#### 2.1.2. PICO Statement

The following PICO (problem/population, intervention, comparison, and outcome) framework was developed:

- Population: domesticated dogs.
- Intervention: exposure to mild-to-moderate pressure, including the use of external pressure, compression wraps, and other devices.
- Comparators: domesticated dogs not exposed to mild-to-moderate pressure or dogs exposed to variable amounts of external pressure.
- Outcomes: primary outcomes include changes in heart rate, cortisol concentrations, and clinical signs associated with anxiety.

#### 2.1.3. Inclusion and Exclusion Criteria

The following inclusion and exclusion criteria were used.

Inclusion criteria:

- Domesticated dogs without age or breed restriction.
- Exposure to mild-to-moderate pressure, including the use of external pressure, compression wraps, and other devices. Devices include but are not limited to commercial products like ThunderShirt, Anxiety Wrap, Weighted Dog Calming Vest, and Honest Paws Calm Vest. Combined treatments could be considered.
- Domesticated (control) dogs not exposed to mild-to-moderate pressure or dogs exposed to variable amounts of external pressure.
- Animals can serve as their own control (e.g., evaluated before and after application of a vest or compression wrap).
- Primary outcomes include changes in a physiological marker (e.g., heart rate, respiratory rate), cortisol or other stress hormone concentrations, clinical signs associated with anxiety, or behavioral endpoints.
- Pre-existing anxiety disorder or those exposed to an anxiety-invoking stimulus (e.g., gunshots, firecracker sounds, recorded thunderstorm sounds).
- Can include owner-reported clinical signs.
- Can be from any year of publication or quality.
- Peer-reviewed publication.
- Any study design including randomized clinical trials; observational studies.
- Must include original data.

Exclusion criteria (reason was recorded):

- Any species other than domesticated dogs.
- Other treatments that do not involve external pressure or compression.
- No concurrent control or relevant outcomes.
- No pre-existing anxiety disorder or exposure to an anxiety-invoking stimulus.
- Studies with incomplete information (e.g., conference abstract, meeting poster).
- Case reports lacking a control.
- No original data (e.g., review).

### 2.2. Search Strategy

The review team initially considered existing systematic reviews to address or help to address its research question. English-language systematic reviews conducted within the last 5 years were sought using searches in PubMed, PROSPERO (CRD), and CAMRADES. No relevant systematic reviews on this topic were identified.

In addition to consideration of systematic reviews, a search for bibliographic references was performed through Cab Abstracts, PubMed, and Web of Science to locate studies. The search was limited to domesticated dogs and performed without sex, age, or breed restrictions. Only peer-reviewed publications in English were considered. The search strategies included descriptors or words in the text related to vests, anxiety, and dogs. The search was developed with input from a librarian (PG) with expertise in the conducting of systematic reviews. A combination of the controlled vocabulary and keywords for the three concepts previously stated (vests, anxiety, dogs) was used to complete the initial literature search. The initial search was performed on 26 October 2023, updated on 24 September 2024, and citations were uploaded into Covidence (www.covidence.org (26 November 2024)).

### 2.3. Study Selection

Screening and quality assessment were tracked in Covidence. The evaluation of titles, abstracts, and the full text was independently performed by a team of two reviewers at both the initial screening (DCD, SM, SS) or full-text review (SM, SS) steps. Reviewers were not involved with any of the reviewed studies. Disagreements were resolved by either discussion or when consensus could not be reached using a third reviewer.

#### 2.3.1. Data Extraction

Extraction of originally published graphical data relied on DigitizeIt version 2.5.1. (Braunschweig, Germany). Data were extracted from included studies by one member of the review team and checked by a second member (SM, SS) for completeness and accuracy. Any discrepancies in data extraction were resolved through discussion. The extracted data were used to summarize study designs and findings. Specific study endpoints that were extracted included spontaneous locomotor activity, mean anxiety score, heart rate, rectal temperature, respiratory rate, skin temperature, activity, globally assessed anxiety scores, and behavioral anxiety symptoms.

#### 2.3.2. Risk of Bias Evaluation

The risk-of-bias domains and questions for assessing risk of bias in experimental studies were based on established guidance for animal studies [30]. The following domains were assessed: blinding of participants and personnel, random selection of animals for outcome assessment, blinding of outcome assessment, incomplete outcome data, selective reporting, and other biases. Experimental studies were independently assessed by two assessors (SM, SS) who answered all applicable risk of bias questions with one of three options (low risk of bias, unclear risk of bias, or high risk of bias) following prespecified criteria (Supplemental Materials). Any discrepancies were resolved through discussion or the use of a third individual. Risk of bias was assessed at the outcome level.

#### 2.3.3. Strategy of Data Synthesis

A narrative synthesis (e.g., study design, year of publication, subject baseline demographics, sample size, country where study was conducted, interventions, and the results from each study) was performed for each outcome.

### 2.4. Animal Use

This systematic review utilized data collected from previous studies. Therefore, ethical approval was not required.

## 3. Results

### 3.1. Results of the Search

**Figure 1:**
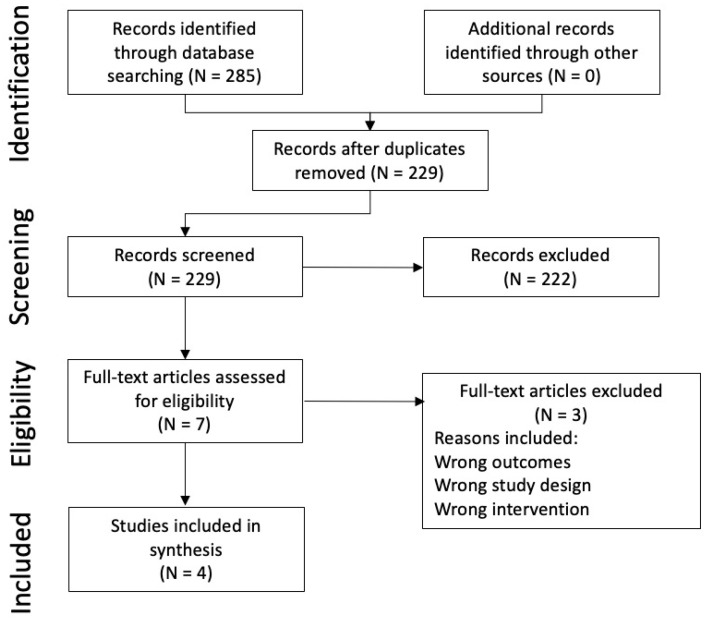
PRISMA diagram.

The search strategy identified 285 citations, of which 56 were duplicate citations. Another 222 citations were excluded based on the title or abstract. The literature was almost entirely identified and retrieved from electronic bibliographic sources. No studies were identified from hand-searching reference lists provided in the studies that met inclusion criteria. A total of seven studies were assessed for inclusion using a review of the full text. The three studies excluded at the full-text review stage, with the reason for exclusion, are provided in Table S1. A total of four studies met the inclusion criteria for this review. A flow diagram for the inclusion of studies in the systematic review is provided in Figure 1.

### 3.2. Reviewed Studies

Four studies met our inclusion criteria [25,26,27,28]. An overview of these studies is provided in Table 1.

Selected demographic characteristics of study populations from the reviewed studies are presented in Table 2.

### 3.3. Risk of Bias

**Figure 2:**
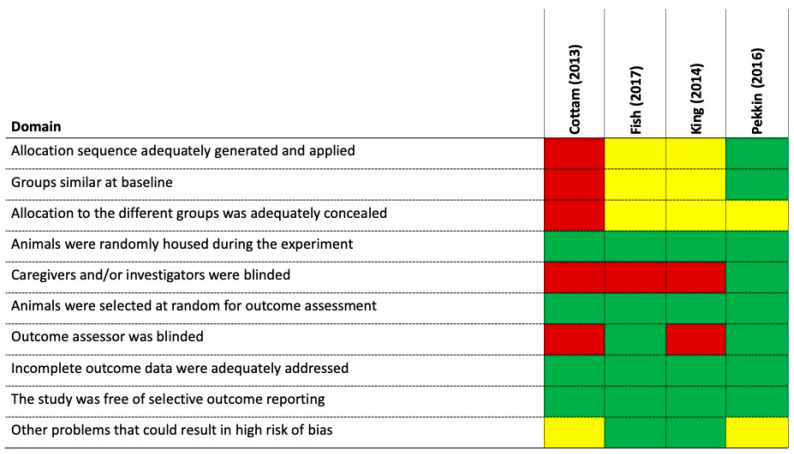
Risk of bias of individual studies. Colors denote low risk of bias (green), unclear risk of bias (yellow), or high risk of bias (red). Cottam [25], Pekkin [26], King [27], Fish [28].

Summary risk-of-bias assessments for the included studies are presented in Figure 2. Critical risk-of-bias domains included groups being similar at baseline, blinding for certain outcomes (e.g., behavioral evaluations), incomplete outcome data, selective reporting, and other sources of bias, including concerns about statistical analyses. Each of the four studies had experimental designs or reporting features that contributed to a high risk of bias in one or more risk-of-bias domains. One study performed by Cottam and coworkers [25] was an open-label trial lacking a placebo or control group. High risk of bias ratings for this study [25] were due to the lack of groups being similar at baseline, missing information on how the dogs were allocated, no concealment of allocation or groups, and investigators not being blinded. This study [25] was funded by the manufacturer of the vests used and their role in the study was undocumented, resulting in an unknown risk of bias.

The study performed by Pekkin and coworkers [26] was double-blinded and all animals were initially semi-randomly divided into three treatment order groups, balanced for gender. Each dog in this study participated in three noise test days with the minimum time between two test days being one week. Each dog thus underwent all three treatments. This study [26] was funded by the manufacturer of the vests used and their role in the study was undocumented, resulting in an unknown risk of bias.

The study performed by King and coworkers [27] randomly assigned dogs to three groups (dogs wore the ThunderShirt per the manufacturer’s recommendations, dogs wore the ThunderShirt loosely without pressure, and a no vest control). Domains with a high risk of bias in this study [27] were the result of investigators and outcome assessors not being blinded to treatment groups.

The high risk of bias for Fish et al., 2017 [28], reflects that the investigators were not being blinded to treatment groups. In this study [28], the outcome assessor evaluating recorded videotapes was unaware of when the recorded thunderstorm sounds were presented as a stimulus. In addition, some physiological measures were collected remotely using the telemetry vest.

## 4. Discussion

This systematic review evaluated whether pressure wraps were an effective anxiolytic for canines with anxiety disorders. Only four studies met our inclusion criteria. Different commercially available products were used in the reviewed studies, including three pressure wraps (Anxiety Wrap, ThunderShirt, Lymed Dog) and one telemetry vest. One of the reviewed studies involved experimental exposure to recorded fireworks sounds for dogs with preexisting fear responses to fireworks [26]. Investigators in this study [26] fitted dogs with vests with different pressure levels (2–3 mm Hg or 10–12 mm Hg). Each dog received all three experimental conditions (no vest, light pressure vest, higher pressure vest) during the three-day experiment. No details were provided concerning how vest pressure was measured or confirmed during the study. Dogs were acclimated to an experimental chamber for approximately 30 min and salivary cortisol measurements taken at the end of this period demonstrated no significant increase in samples collected immediately prior to entry into the room. Each dog’s owner was also present in the experimental room during this test; however, they were kept separated from their dog by a one-meter-tall fence. The dog’s behavior in the experimental chamber was video-monitored during three sequential two-minute intervals (pre-noise, firework sound, recovery) that occurred near the end of the test session. Use of the higher-pressure vest was associated with decreased lying time during the two-minute noise interval when compared with controls who were not wearing a vest. Overall, the authors of this study [26] concluded that they “did not find a clear therapeutic effect of using pressure vests in noise phobic dogs”.

Functional magnetic resonance imaging studies in humans have shown that intranasal administration of oxytocin reduced activation of the amygdala and reduced coupling of the amygdala to brainstem regions implicated in fear responses [31]. In dogs, positive interactions with owners have been shown to increase blood oxytocin concentrations in both the owner and the dog [32]. Oxytocin also has additional effects on blood pressure and heart rate [33]. The impact of vest-wearing on urine oxytocin concentration was evaluated in one study [26]. However, urine oxytocin concentrations were only determined one week prior to the study start and occurred before fitting the vest and after the vest was worn for 30 min. A significant association was seen between urinary oxytocin concentration after wearing the higher pressure (10–12 mm Hg) vest for 40 min and owner-reported general fearfulness, noise fear frequency, and reactivity index [26]. This study also evaluated saliva cortisol concentrations after the dog arrived at the laboratory, just prior to the noise stimuli and 20 and 40 min after the end of the presentation of the noise stimuli. Saliva cortisol levels measured at 20 min after noise exposure were higher than those seen prior to exposure to the nose as well as 20 min later. No significant associations between saliva cortisol levels and questionnaire-derived behavioral variables were seen in this study. The results of this study suggest that the measurement of urinary oxytocin and saliva cortisol concentrations may be of value in the evaluation of pressure wraps as an anxiolytic in dogs.

A second experimental study [28] exposed dogs with unknown noise sensitivity to recorded thunderstorm sounds. This study assessed the effect of a telemetry vest on behavioral and physiological responses in Labrador retrievers exposed to recorded thunderstorm sounds. Dogs were held in a 7.8 m^2^ room for nine minutes on three consecutive days. The nine-minute test period was divided into three-minute test phases. The first and last three-minute test phases on each day were quiet. The middle test phase was either quiet on days one and three or included a three-minute recording of a thunderstorm on day two. Video analysis of motor activity and anxiety-related behavior and manual determination of heart rate and body temperature were performed. Dogs in the control (no vest group) were fitted with the telemetry undershirt and vest prior to the open-field test to simulate handling procedures used in the vest group. The undershirt and vest remained on for approximately 2 min while the investigators obtained manual heart rate and rectal temperature. Vests and undershirts were removed immediately prior to the start of the open field test; therefore, these video analyses were not blinded to vest wearing. These analyses were blinded to the session (no noise, thunderstorm noise). Heart rate and respiratory rate were also collected using telemetry during the open field test. Vest wearing did not affect total locomotor activity or rectal body temperature but significantly decreased heart rate by 8% and overall mean anxiety score by 34% during open-field test sessions where recorded thunderstorm sounds were used as a stimulus.

Both reviewed experimental studies that used recorded loud noises [26,28] have important limitations. Noise levels used in the fireworks and thunderstorm studies were approximately 70 and 90 dB, respectively. These sound levels are lower than what a dog could be exposed to since both thunder and firework sounds can exceed 100 dB depending upon the dog’s proximity to the noise source [34,35]. Both reviewed studies that used recorded loud noises [26,28] presented the loud noise stimulus for only two to three minutes, while naturally occurring thunderstorms and firework displays can last significantly longer. Since longer exposure periods might yield different physiological or behavioral outcomes, future studies using longer duration and intensity sounds are warranted.

A third clinical study evaluated the effectiveness of a pressure wrap in reducing thunderstorm phobia [25]. This open-label study recruited owners of dogs with thunderstorm phobia. Participating owners completed a questionnaire regarding the intensity, frequency, and duration of behaviors observed during a thunderstorm. Owner reports of these behaviors were used to calculate anxiety scores. Questionnaires were completed for two thunderstorm events prior to the use of a pressure wrap (baseline) and then during five subsequent thunderstorm events while their dog was wearing a pressure wrap. This open-label study [25] reported that mean anxiety scores were decreased during the fifth thunderstorm event when compared with baseline anxiety scores. Most (89%) owners reported that the pressure wrap was at least partially effective in managing their dogs’ thunderstorm phobia. Eighty percent of the owners reported that they would continue to use the Anxiety Wrap for their dogs’ thunderstorm phobia after the trial. This study has several important limitations, including the absence of an external control group. Owners were aware of the treatment, which could contribute to a placebo effect. Indeed, Cottam and Dodam (2009) hypothesized that a placebo effect may have accounted in part for some of the benefits seen with the use of an anti-static cape in dogs with thunderstorm phobia [24]. In addition, the act of scoring behaviors during thunderstorms may alter an owner’s behavior with subsequent impacts on their dogs’ behavior as well.

The remaining reviewed experimental study evaluated the use of a pressure wrap (ThunderShirt) on heart rate and behavior in dogs diagnosed with either separation anxiety or generalized anxiety disorder when isolated from their owner in a kennel for 15 min [27]. Wearing a tightly fitted ThunderShirt was associated with a lower elevation in average heart rate following isolation in a kennel when compared with dogs fitted with either a loose pressure wrap or no wrap. Dogs wearing a tightly fitted ThunderShirt who were not receiving anxiety medication had a lower maximum heart rate when compared with non-vest-wearing controls. Dogs in the control group spent more time orienting toward the kennel door when compared with dogs wearing a vest. This test was performed in an unfamiliar location, which may also have altered the dog’s response to the separation and influenced the impact of vest-wearing.

One limitation of the three reviewed experimental studies concerns the duration of the anxiety-invoking stimulus. Studies that used recorded loud noises [26,28] presented the loud noise stimulus for only two to three minutes. Another study that evaluated separation anxiety [27] relied on separating the dog from their owner for a relatively short (15 min) time. The duration of the anxiety-evoking stimuli may not mimic real-world experiences in a home environment. Despite this limitation, a reduction in behavioral or physiological signs of anxiety under the compression wrap condition would still provide evidence of a benefit in a study using short-term exposure to a stressor.

Another factor that needs to be considered is the amount of pressure generated by a pressure wrap. Two of the reviewed studies [26,27] included variable pressure wrap intensities. The results from both studies provide some evidence that the efficacy of vest-wearing is enhanced when vest pressure is increased, resulting in a tightly fitted garment. Similar findings have been reported with the use of swaddling human infants where some evidence suggests that tight swaddling may be more effective at increasing the duration of quiet sleep and reducing the number of sleep state changes in infants [36]. Additional insights into the use of pressure in the management of anxiety syndromes can be gleaned from several recent systematic reviews evaluating the use of weighted blankets in reducing anxiety [37,38] or mental disorders [39] in humans. The authors of these systematic reviews noted a high risk of bias, primarily due to the failure to blind participants, for participant-reported measures. The authors also noted the relative paucity of studies (fewer than ten in each review), small numbers of participants, and a lack of standardization of the blanket weight as factors that contributed to the heterogeneous results seen. One of the systematic reviews evaluating weighted blankets [37] found that the use of a 14-pound or 20-pound weighted blanket or a 5-pound lap pad for approximately 20 min significantly decreased anxiety and pulse rate in adults experiencing anxiety in an inpatient mental health unit. This study [37] also reported that the reduction in anxiety was independent of the weight of the blanket used, suggesting a variable response to increasing pressure. The use of a pressure wrap could also produce discomfort in a patient. Reported pleasantness ratings on the wrist, forearm, bicep, ankle, and calf fitted with an inflatable sleeve were generally rated highest by people when pressure pulses of 50 or 70 mmHg were applied [40].

Despite the potential importance of pressure vest tightness, few of the reviewed studies attempted to measure or control it, weakening the value of the studies reviewed. Moreover, the amount of tactile pressure to the torso needed to induce a calming effect on the nervous system in dogs remains unknown. Future studies evaluating the pressure generated by these garments on dogs of various sizes and body condition scores would provide useful information for the design of future experimental studies. Several different approaches are available to measure pressure exerted by a garment, including the use of pressure transducers [41,42].

Each of the studies had additional limitations affecting the quality of the studies and their results. All reviewed studies [25,26,27,28] had a relative lack of blinding, leading to possibly biased results, meaning the reported effectiveness of the pressure wrap as an anxiolytic may be incorrect. In addition, one study [25] lacked a control or historical data as comparators to the experimental groups. This makes it difficult to determine whether the use of a pressure wrap improved dogs’ anxiety symptoms. Several reviewed studies [25,26,27] relied on owner-reported outcomes. Because it was impossible to blind the owner to the treatment, the owners could be unconsciously biased to the effect the wrap had on their dog, causing them to report outcomes that may be incorrect or exaggerated. Ideally, a double-blind and placebo-controlled study should be conducted to adequately assess the therapeutic value of pressure wraps in the treatment of canine anxiety; however, the development of a suitable placebo remains challenging.

Our study summarizes the scant available literature and therefore has several important limitations. Only four studies met our inclusion criteria and outcomes varied amongst the four studies. The interventions and stimulus used also varied across the studies reviewed, and the interpretation and transferability of the results should be treated with caution. It would be beneficial to include a wider variety of results from more studies with lower risks of bias to make a more reliable and accurate conclusion about the effectiveness of pressure wraps as an anxiolytic for dogs with anxiety disorders. Another limitation is that advanced registration of the study protocol did not occur. In the past few years, one registration vehicle, PROSPERO4animals, has emerged [43]. Pre-registration of a systematic review protocol strictly follows best practices developed by the scientific community. Pre-registration helps minimize bias in the conduct and reporting of the systematic review and helps reduce duplication of effort between groups [44]. Another limitation was that the original searches were performed using English as the publication language. This can introduce bias into a systematic review [45,46]. To address this concern, the authors also evaluated whether any non-English publications were available in PubMed. This search was conducted immediately prior to the publication of this study and yielded no additional non-English studies that met our inclusion criteria.

Our study identified a need for future research on these products. Robust, experimental studies using cross-over designs or vests with different pressures are needed to evaluate the available products. Since the available commercial products have different design features and textiles, product-specific studies will be needed for each garment. Sham garments that mimic the overall design of a commercially available product but produce different pressure profiles may be needed in some studies. Future studies would also benefit from the measurement of the pressures being produced by the test garment. Future studies would also benefit from the inclusion of additional objective measures, including biomarkers of stress responses. The activity levels of animals enrolled in future studies could be assessed using accelerometers and other approaches. Heart rates could also be assessed using optical heart sensors and related technologies.

## 5. Conclusions

Our systematic review indicates that pressure wraps for the management of canine anxiety are associated with short-term reductions in either a physiological marker of stress (e.g., heart rate) or an improvement in anxiety symptoms. Experimental studies performed to date have important limitations, including a relative lack of blinding and short-term exposure (minutes) of dogs to anxiety-invoking stimuli that may not mimic real-world conditions where stressor duration can be much longer (hours). Only one open-label study evaluated the effect of the pressure wrap on natural stimuli in the home environment. Most owners reported that the pressure wrap was at least partially effective in managing their dogs’ thunderstorm phobia and that they intended to continue its use after the completion of the trial. However, this open-label study relied on owner evaluations, which may be skewed by unaccounted-for placebo effects. An advantage of pressure wraps for the management of canine anxiety is the lack of reported side effects associated with the use of these devices. Our review suggests there is weak and limited evidence to support the beneficial effect of pressure wraps in reducing physiological or behavioral signs of anxiety in response to an anxiety-invoking stimulus. Confidence in the available literature is low due to moderate-to-high risks of bias and inconsistent findings. Robust, blinded, and well-powered future studies are needed to make more reliable and accurate conclusions regarding the efficacy of these products.

## Figures and Tables

**Table 1:** Reviewed studies that evaluated the use of pressure wraps on sound-or separation-induced anxiety. Abbreviations: d: day; dB SPL: decibel sound pressure level.

Study	Study Description	Outcome of Interest	Main Findings	Comments
Cottam et al., 2013 [25]	Open-label experimental study evaluating the effectiveness of repeated (up to five) applications of a commercially available pressure wrap (Anxiety Wrap) on naturally occurring canine thunderstorm phobia. Owners put on the wrap three times when thunderstorms were absent to reduce the association of the wrap with storms (baseline). Owners used the wrap and filled out surveys during five subsequent thunderstorm events.	Owner-reported Thunderstorm Anxiety Scores before and after the use of the Anxiety Wrap. Owners assessed presence and severity of nine behaviors (pant, shake, inappropriate elimination, pace, attention seeking, vocalization, inappetence, salivation, hiding) using a five-point Likert scale. These values were used to calculate anxiety scores at baseline (twice) and during treatment during five thunderstorm events. A post-treatment survey evaluating the owner’s impression of the effectiveness of the wrap was also completed.	The mean Thunderstorm Anxiety Score associated with the fifth use of the Anxiety Wrap was 47% lower than the initial mean anxiety score. There was a significant increase in the number of owners who rated the wrap effective (*n* = 17) versus the number of owners who rated the wrap ineffective (*n* = 2). There was a significant decrease in the percentage of owners reporting pacing and shaking in dogs wearing a vest. The majority (89%) of owners reported that the Anxiety Wrap was at least partially effective in treating their dogs’ thunderstorm phobia. Most (80%) owners reported that they would continue to use the Anxiety Wrap for their dog’s thunderstorm phobia after the end of the trial. Negative side effects were not reported.	No placebo/control group was included. The vest was tightly wrapped around each dog’s torso and could be dampened with water if the owner was worried their dog would overheat. Owners practiced fitting the wrap once before a thunderstorm to associate it with a reward. Owners were not blinded to their treatment. All dogs received the same treatment. Study funded by Animals Plus LLC, Huntington, IN.
Fish et al., 2017 [28]	Randomized and placebo-controlled experimental study evaluating the effect of a Lomir undershirt and telemetry vest (Lomir Biomedical, Quebec, Canada) on behavioral and physiological parameters of Labrador retrievers in response to an environmental stressor (recorded thunderstorm sounds). The telemetry was tightened to allow two fingers to be placed under the vest. Dogs in the no vest group were fitted with the telemetry undershirt and vest prior to the open field test to simulate handling procedures used in the Vest group. The undershirt and vest were used to obtain manual heart rate and rectal temperature (approximately 2 min) and were removed immediately prior to the start of the open field test.	Spontaneous locomotor activity, mean anxiety score, heart rate, rectal temperature, skin temperature, and activity. Evaluation of recordings collected during the open field test were performed without sound by an individual who was unaware of whether recorded thunderstorm sounds were present.	The mean anxiety score during the thunderstorms decreased 34% in the treatment group (vest: 95.5 ± 1.5 bpm: control: 103.9 ± 2.0 bpm). Heart rate decreased by 8% in the treatment group. There was no effect on spontaneous motor activity. Negative side effects were not reported.	Prior to the study, global anxiety scores were used to rank the dogs from lowest to highest anxiety rating. The first of each pair of dogs was randomly assigned to either Vest or no vest groups (*n* = 8/group). There was no significant difference in global anxiety scores for the two experimental groups. Open field test for 9 min on three consecutive test days: days 1 and 3: 9 min no auditory stimuli; day 2: 3 min no auditory stimuli, 3 min audio recording of a thunderstorm, 3 min no auditory stimuli. The mean thunderstorm sound level was 88.8 dB SPL; the peak level was 104 to 105 dB; the A-weighted sound exposure level was 110.9 dBA. Study funded by K2 Solutions and the United States Office of Naval Research.
King et al., 2014 [27]	Randomized experimental study investigating the use of a commercially available pressure wrap (ThunderShirt) on heart rate and behavior in dogs with separation anxiety or generalized anxiety disorder. Study compared wearing the vest according to the manufacturer’s instructions (tight) vs. wearing the vest with no pressure (draped) vs. no vest.	Heart rate and behavioral anxiety signs were assessed in an experimental kennel following separation from their owner. Dogs were isolated in a research kennel away from their owners for 15 min. Baseline heart rate measurement was taken prior to separation from the owners.	Dogs separated from their owner that wore the Thundershirt tightly had significantly less of an increase from baseline in the average heart rate when compared with either control (no vest) or dogs wearing a loosely fitted vest. Dogs that wore the ThunderShirt tightly did not differ significantly from controls in maximum heart rate when all dogs were considered but did differ significantly from the controls when only those dogs not currently on anxiety medication were considered. Dogs in the control group were more likely to orient towards the door than the dogs wearing a pressure wrap. Other behavioral outcomes were unaffected by vest wearing. Negative side effects were not reported.	The dogs were randomly assigned to vest, loose vest, or no vest groups (*n* = 30/group). Investigators reviewed video recordings and noted the presence or absence of calm behaviors. ANCOVA was used to assess differences in heart rates. Owners were not blinded to the treatment. Self-funded study.
Pekkin et al., 2016 [26]	Double-blinded experimental study determining if a commercially available pressure vest (Lymed Animal) had a beneficial effect on the behavior of noise-phobic dogs when exposed to recorded firework sounds. Two pressure conditions, approximately 10–12 mmHg (DEEP) and approximately 2–3 mmHg (LIGHT), and a no vest control were used as treatments. There were three test days when noise was used. The test period was divided into three two-minute intervals (pre-noise quiet interval, noise interval, and a quiet recovery interval). Noise was provided by recorded firework sounds (70–73 dB). Owners were seated in the experimental room but separated from the dog by a short fence.	Activity, body and tail postures, vocalization, and time spent near owner were assessed. Behaviors were videorecorded during the 6 min test period. Urine oxytocin concentrations were measured prior to the start of the study (after initial fitting and after wearing the vest for 30 min). Salivary cortisol concentrations were measured prior to the start of the noise tests and at 20 and 40 min post-noise.	Salivary cortisol concentrations in samples collected 20 min after the end of the 2 min sound stimuli were 15 to 25% higher versus samples collected prior to the sound stimuli or collected 40 min after the end of the 2 min sound stimuli. Total time spent lying down during the noise interval with either pressure vest correlated positively with the 20-min post-noise saliva cortisol concentration. A significant association was seen between urinary oxytocin concentration after wearing the higher pressure (10–12 mm Hg) vest for 40 min and owner-reported general fearfulness, noise fear frequency, and reactivity index. The time spent near the owner during the recovery interval during the DEEP treatment correlated positively with urine oxytocin concentrations). A significant decrease in time lying down during noise stimuli was seen (DEEP versus control). Lying duration during the noise interval was positively correlated with saliva cortisol concentration when wearing either vest. Wearing the DEEP vest increased the time the dogs spent near their owner during the noise and recovery intervals. Negative side effects were not reported. Owners reported mainly positive or neutral experiences when exposure to firework noise may have occurred.	Each dog underwent all three treatments (control, LIGHT, DEEP). Missing data are addressed; altogether, entire data from physical and behavioral measures were available from 20 dogs. Owners completed a survey prior to the experiment and a follow-up questionnaire afterwards. Urinary oxytocin concentrations were collected in the absence of anxiety-invoking stimuli, so they were deemed a less relevant outcome in this review. No details were provided concerning how pressure was measured or confirmed during the study. Study funded by Lymed Oy, Alma and K.A. Snellman Foundation, Finnish Foundation of Veterinary Research, and the European Research Council.

**Table 2:** Select demographic data for the reviewed studies. Abbreviations: CM: castrated male; F: female; M: male; mo: month; NR: not reported; y: year.

Study	Breed	Sex and Number	Age Range	Pre-Study Status
Cottam et al., 2013 [25]	NR	*n* = 18 (completed all phases; *n* = 21 baseline)	2.7 to 7.6 y	Dogs displayed at least three out of ten anxious behaviors (panting, shaking, escaping attempts/property destruction, inappropriate elimination, pacing, attention seeking, whining, inappetence, salivation, and hiding) during a thunderstorm to be eligible. Subjects displayed anxiety for ≥85% of the time during a thunderstorm. Non-house trained dogs, dogs with pre-existing health conditions, and dogs undergoing pharmacological treatment for thunderstorm phobia ineligible for enrollment.
Fish et al., 2017 [28]	Labrador retriever	M (8), 5 F (5), 3 SF (3)	2.50 to 4.25 y	Dogs were used in prior studies to assess their emotional resilience and visual and olfactory discrimination capacities. All dogs had previous exposure to the telemetry system and open-field test approximately 4 and 6 months prior to the conduct of the study, respectively.
King et al., 2014 [27]	No breed restrictions: Terrier, Herding, Toy, Working, Sporting, Non-sporting, Hound	M (39), F (51)	>0.5 y	Included dogs (>6 mo of age) were diagnosed with either separation anxiety or generalized anxiety disorder. Dogs with any other health issues were excluded. No other pre-study inclusion criteria were given.
Pekkin et al., 2016 [26]	Lagotto Romagnolo (7), Staffordshire Bullterriers (6) most frequent breeds	M (5), CM (5), F (4), SF (14)	2.0 to 11.0 y	Dogs recruited via an ongoing study investigating genetic background of noise sensitivity. Dogs fearful towards people or new situations as well as female dogs in estrus and dogs using regular medication were excluded except for dogs using non-steroidal anti-inflammatory drugs.

## Data Availability

All data have been provided.

## Supplementary Materials

The following supporting information can be downloaded at: https://www.mdpi.com/article/10.3390/ani14233445/s1, Table S1: List of studies that were excluded based on a review of the full text. The reason for exclusion is also provided [47,48,49,50].
