# Supplementary material for: A Systematic Review of the Efficacy of Compression Wraps as an Anxiolytic in Domesticated Dogs
Source: Animals (Basel). 2024 Nov 28;14(23):3445. doi: 10.3390/ani14233445 (PMC11639916; doi:10.3390/ani14233445)
Supplement: Supplementary file 1 [file animals-14-03445-s001.zip › animals-3313554-supplementary.pdf]

Supplementary Material

A Systematic Review of the Efficacy of Compression Wraps as an Anxiolytic in Domesticated Dogs

BACKGROUND AND INTRODUCTION

Canine anxiety disorders are common behavior problems seen in veterinary clinics and hospitals across the United States. Treatments for these disorders incorporate medication, behavior modification programs, and alternative techniques. Moderate-to-deep pressure has been used in animals to reduce tension and anxiety (Diego and Field, 2009, Edelson et al., 1999, Williams and Borchelt, 2003).

Several studies have examined the use of pressure (e.g., the use of weighted materials, capes or wraps) in canines in reducing aggression (Williams & Borchelt, 2003), response to thunderstorm noise (Cottam and Dodman, 2009; Cottam et al., 2013), firework sounds (Pekkin et al., 2016), or anxiety disorders (King et al., 2014). Our laboratory has shown that dogs wearing telemetry vests had reduced anxiety when presented with thunderstorm sounds (Fish et al., 2017).

The aim of the present study was to determine whether there was an effect of a pressure wrap on either physiologic measures (e.g., heart rate) or behavior in domesticated dogs presented with an anxiety-inducing response or previous diagnosis with an anxiety disorder.

OBJECTIVE AND SPECIFIC AIMS

Review Question: Does mild to moderate pressure reduce either behavioral or physiological markers of anxiety in dogs with either pre-existing anxiety disorder or those exposed to an anxiety invoking stimulus.

The specific aims of the first review are to:

- Identify literature reporting the use of pressure on domesticated dogs as an anxiolytic treatment.
- Extract data on anxiolytic effects of pressure from relevant studies.
- Assess the internal validity (risk of bias) of individual studies.
- Summarize the extent of evidence available.
- Synthesize the evidence using a narrative approach or meta-analysis (if appropriate) considering limitations on data integration, such as study-design heterogeneity.
- Rate the confidence in the body of evidence for studies.

PICO STATEMENT

- Population: Domesticated dogs.
- Intervention: Exposure to mild to moderate pressure including the use of external pressure, compression wraps, and other devices.
- Comparators: Domesticated dogs not exposed to mild to moderate pressure or dogs exposed to variable amounts of external pressure.
- Outcomes: Primary outcomes include changes heart rate, cortisol concentrations, clinical signs associated with anxiety.

METHODS

<u>Problem Formulation and Protocol Development:</u> The review question and specific aims were developed and refined through a series of problem formulation steps.

<u>Review Team</u>: The review team at North Carolina State University College of Veterinary Medicine will include David Dorman (DCD; veterinary toxicologist), Margaret Gruen (MG; veterinary behaviorist), Peggy Gross (PG: medical librarian), and Savannah Mathis (SM; student). If a member of the review team was a coauthor of a study under review, that member will recuse himself or herself from the evaluation of the quality of that study. The review team will be responsible for performing all aspects of the review, including conducting the literature searches; applying inclusion/exclusion criteria to screen studies; extracting data; assessing risk of bias for included studies; and analyzing and synthesizing data. The roles and responsibilities of the team members will be documented throughout the protocol. Throughout the course of its work, the review team will also engage others as needed. The involvement of those individuals will be documented.

Search Methods

- Literature Search for Independent Systematic Review
    - An information specialist (PG) who has training, expertise, and familiarity with developing and performing systematic review literature searches will design the search. A variety of methods will be used to identify relevant data (see below). Literature searches will not be limited by publication date.
    - Online Databases
        - Electronic searches of the following three online databases will be performed: PubMed and Web of Science. The search strategy and search terms will be developed by the information specialist (PG), who will implement the search for relevant studies.
    - Other Resources
        - Hand searching the reference lists of all the included studies after full-text review will be conducted using the same study selection process as used for screening records retrieved from the electronic search. Relevant studies identified through these steps will be marked as "provided from other sources" in the study selection flow diagram.
        - Study Selection
            - All search results will be imported or manually entered into Covidence or another reference management software.
            - Covidence will be used to eliminate any duplicate citations before evaluating the eligibility of the citations.

Screening Process

- References retrieved from the literature search will be screened for relevance and eligibility against the evidence selection criteria using Covidence (https://www.covidence.org/home). Screeners from the review team (DCD and MS) will be trained with an initial pilot phase on several studies undertaken to improve clarity of the evidence selection criteria and to improve accuracy and consistency among screeners.
- Title and Abstract Screening
    - Title and Abstract Screening will be initially performed followed by Full-Text Screening of studies that are initially identified.
    - At each step, each citation will be independently screened by two reviewers (DCD and MS) to determine whether it meets the selection criteria for inclusion that reflect the PICO statement with some additional considerations as listed below. Citations included at the title/abstract screening level will be subject to a full-text review by the same two reviewers. Disagreements regarding citation eligibility will be resolved via consensus and, where necessary, by consulting a committee member.
    - Exclusion criteria: The title/abstract screening form will be used to screen and EXCLUDE references if at least one of the following criteria is met:
        - No original data (e.g., review article, commentary, editorial)
        - Study does not include domesticated dogs
        - Study does not report use of external pressure
        - No relevant outcomes
        - Incomplete information (e.g., conference abstract, meeting poster)
        - Other (explanation required)
    - Inclusion criteria: The following types of records will be INCLUDED at the title/abstract level:
        - No language restriction

Data Extraction

- Data will be collected and recorded (i.e., extracted) from included studies by one member of the review team and checked by a second member for completeness and accuracy. Any discrepancies in data extraction will be resolved through discussion. The extracted data will be used to summarize study designs and findings and/or to conduct statistical analyses. Data extraction elements that will be used will be documented.
- Data extraction will be completed using study specific tables.
- Data extraction elements will include:
    - Funding
        - Funding source(s)
        - Reporting of COI by authors
    - Animal Model
        - Sex
        - Source of animals
        - Age or life stage at start of dosing and at health outcome assessment
    - Treatment
        - Source of pressure used
        - Duration and frequency of pressure use (e.g., hours, days, weeks when administration was ended, days per week)
        - Intensity of pressure

- Methods
  - Study design
  - Number of animals per group
  - Randomization procedure, allocation concealment, blinding during outcome assessment
  - End point health category (e.g., behavior, physiologic)
  - End point (e.g., heart rate, cortisol concentration, behavior, motor activity)
  - Diagnostic or method to measure end point
  - Statistical methods
- Results
  - Measures of effect (e.g., mean, median, frequency, and measures of precision or variance) or description of qualitative results.
  - Statistical significance.
- Other
  - Use of digital rulers to estimate data values from figures, exposure unit, and statistical result conversions, etc.

Risk of Bias (Quality) Assessment of Individual Studies

- Risk of bias is related to the internal validity of a study and reflects study-design characteristics that can introduce a systematic error (or deviation from the true effect) that might affect the magnitude and even the direction of the apparent effect. The risk of bias domains and questions for experimental animal studies are based on established guidance for experimental human studies (randomized clinical trials) (Higgins and Green 2011; Viswanathan et al. 2012, 2013; Sterne et al. 2014) and recent tools for animal studies (Hooijmans et al. 2014; Koustas et al. 2014). The risk of bias tool includes a common set of questions that are answered based on the specific details of individual studies to develop risk of bias ratings (using the four options: definitely low risk of bias; probably low risk of bias; probably high risk of bias; or definitely high risk of bias).
- Information or study procedures that were not reported are assumed not to have been conducted, resulting in an assessment of "probably high" risk of bias.
- Studies will be independently assessed by two assessors who answer all applicable risk of bias questions with one of four options following prespecified criteria. The criteria describe aspects of study design, conduct, and reporting required to reach risk of bias ratings for each question and specify factors that can distinguish among ratings (e.g., what separates "definitely low" from "probably low" risk of bias). Risk of bias will be assessed at the outcome level because study design or method specifics may increase the risk of bias for some outcomes and not others within the same study. Information or study procedures that were not reported are assumed not to have been conducted, resulting in an assessment of "probably high" risk of bias.
- Assessors (DCD and SM) will be trained using the criteria in an initial pilot phase undertaken to improve clarity of criteria that distinguish between adjacent ratings and to improve consistency among assessors. All team members involved in the risk of bias assessment will be trained on the same set of studies and asked to identify potential ambiguities in the criteria used to assign ratings for each question. Any ambiguities and rating conflicts will be discussed relative to opportunities to refine the criteria to more clearly distinguish between adjacent ratings. If major changes to the risk of bias criteria are made based on the pilot phase (i.e., those that would likely result in revision of response), they will be documented in a protocol amendment along with the date modifications were made and the logic for the changes. It is also expected that information about confounding, exposure characterization, outcome assessment, and other important issues may be identified during or after data extraction, which can lead to further refinement of the risk of bias criteria.
- After assessors have independently made risk of bias determinations for a study across all risk of bias questions, the two assessors will compare their results to identify discrepancies and attempt to resolve them. Any remaining discrepancies will be considered and resolved with another member of the review team (MG). The final risk of bias rating for each question will be recorded along with a statement of the basis for that rating.

RISK OF BIAS ASSESSMENT

Domain = Selection bias: occurs when individuals or groups in a study differ systematically from the population of interest leading to a systematic error in an association or outcome.

1. Was administered dose or exposure level adequately randomized?

Definitely Low Risk of Bias	Direct evidence that animals were allocated to any study group including controls using a method with a random component, AND there is direct evidence that the study used a concurrent control group as an indication that randomization covered all study groups. Note: Acceptable methods of randomization include: referring to a random number table, using a computer random number generator, coin tossing, or shuffling cards (Higgins and Green, 2011). Restricted randomization (e.g., blocked randomization) to ensure that particular allocation ratios will be considered low bias. Similarly, stratified randomization

	approaches that attempt to minimize imbalance between groups on important prognostic factors (e.g., body weight) will be considered acceptable.
Probably Low Risk of Bias	Indirect evidence that animals were allocated to any study group including controls using a method with a random component (i.e., authors state random allocation, without description of method), AND evidence that the study used a concurrent control group as an indication that randomization covered all study groups, OR it is deemed that allocation without a clearly random component would not appreciably bias results.
Probably High Risk of Bias or (NR)	Indirect evidence that animals were allocated to study groups using a method with a nonrandom component, OR indirect evidence that there was a lack of a concurrent control group, OR there is insufficient information provided about how animals were allocated to study groups (record "NR" as basis for answer).
Definitely High Risk of Bias	Direct evidence that animals were allocated to study groups using a nonrandom method, including judgment of the investigator, the results of a laboratory test, or a series of tests, OR direct evidence that there was a lack of a concurrent control group.

2. Was allocation to study groups adequately concealed?

Definitely Low Risk of Bias	Direct evidence that at the time of assigning study groups the research personnel did not know what group animals were allocated to, and it is unlikely that they could have broken the blinding of allocation until after assignment was complete and irrevocable. Acceptable methods used to ensure allocation concealment include sequentially numbered treatment containers of identical appearance or equivalent methods.
Probably Low Risk of Bias	Indirect evidence that at the time of assigning study groups the research personnel did not know what group animals were allocated to and it is unlikely that they could have broken the blinding of allocation until after assignment was complete and irrevocable, OR it is deemed that lack of adequate allocation concealment would not appreciably bias results.
Probably High Risk of Bias or (NR)	Indirect evidence that at the time of assigning study groups it was possible for the research personnel to know what group animals were allocated to, or it is likely that they could have broken the blinding of allocation before assignment was complete and irrevocable, OR there is insufficient information provided about allocation to study groups (record "NR" as basis for answer).
Definitely High Risk of Bias	Direct evidence that at the time of assigning study groups it was possible for the research personnel to know what group animals were allocated to, or it is likely that they could have broken the blinding of allocation before assignment was complete and irrevocable.

3. Were the research personnel blinded to the study group during the study?

Definitely Low Risk of Bias	Direct evidence that the research personnel were adequately blinded to study group, and it is unlikely that they could have broken the blinding during the study. Methods used to ensure blinding include central allocation; sequentially numbered treatment containers of identical appearance; sequentially numbered animal cages; or equivalent methods.
Probably Low Risk of Bias	Indirect evidence that the research personnel were adequately blinded to study group, and it is unlikely that they could have broken the blinding during the study, OR it is deemed that lack of adequate blinding during the study would not appreciably bias results. This would include cases where blinding was not possible but research personnel took steps to minimize potential bias.
Probably High Risk of Bias or (NR)	Indirect evidence that the research personnel were not adequately blinded to study group, OR there is insufficient information provided about blinding to study group during the study (record "NR" as basis for answer).
Definitely High Risk of Bias	Direct evidence that the research personnel were not adequately blinded to study group.

4. Were experimental conditions identical across study groups?

Definitely Low Risk of Bias	Direct evidence that the same aqueous vehicle was used in control and experimental animals, AND direct evidence that non-treatment-related experimental conditions were identical across study groups (i.e., the study report explicitly provides this level of detail).
Probably Low Risk of Bias	Indirect evidence that the same aqueous vehicle was used in control and experimental animals, OR it is deemed that the aqueous vehicle used would not appreciably bias results, AND identical non-treatment-related experimental conditions are assumed if authors did not report differences in housing or husbandry.
Probably High Risk of Bias or (NR)	Indirect evidence that the aqueous vehicle differed between control and experimental animals, OR authors did not report the aqueous vehicle used (record "NR" as basis for answer), OR there is indirect evidence that non-treatment-related experimental conditions were not comparable between study groups.

Definitely High Risk of Bias	Direct evidence from the study report that control animals were untreated, or treated with a different vehicle than were experimental animals, OR there is direct evidence that non-treatment-related experimental conditions were not comparable between study groups.

5. Can we be confident in the exposure characterization?

Definitely Low Risk of Bias	Information is provided to allow discrimination between exposure groups AND the use of pressure treatment was consistently administered (i.e., with the same method and time frame) across treatment groups and individual animals.
Probably Low Risk of Bias	Information is provided to allow discrimination between exposure groups
Probably High Risk of Bias or (NR)	Indirect evidence that the exposure to pressure was inadequately assessed, OR exposure was inconsistently administered across treatment groups.
Definitely High Risk of Bias	Direct evidence that the exposure to pressure was not assessed OR exposure was inconsistently administered across treatment groups.

6. Blinding during the outcome assessment?

Definitely Low Risk of Bias	Direct evidence that the outcome assessors were adequately blinded to the study group, and it is unlikely that they could have broken the blinding prior to reporting outcomes.
Probably Low Risk of Bias	Indirect evidence that the outcome assessors were adequately blinded to the study group, and it is unlikely that they could have broken the blinding prior to reporting outcomes, OR it is deemed that lack of adequate blinding of outcome assessors would not appreciably bias results, which is more likely to apply to objective outcome measures.
Probably High Risk of Bias or (NR)	Indirect evidence that it was possible for outcome assessors to infer the study group prior to reporting outcomes without sufficient quality control measures, OR there is insufficient information provided about blinding of outcome assessors (record "NR" as basis for answer).
Definitely High Risk of Bias	Direct evidence for lack of adequate blinding of outcome assessors, including no blinding or incomplete blinding without quality control measures.

7. Can we be confident in the outcome assessment?

Definitely Low Risk of Bias	Direct evidence that the outcome was assessed using well-established methods, AND assessed at the same time after initial exposure in all study groups.
Probably Low Risk of Bias	Indirect evidence that the outcome was assessed using acceptable methods (i.e., deemed valid and reliable), AND assessed at the same time after initial exposure in all study groups, OR it is deemed that the outcome assessment methods used would not appreciably bias results.
Probably High Risk of Bias or (NR)	Indirect evidence that the outcome assessment method is an insensitive instrument, OR the length of time after initial exposure differed by study group, OR there is insufficient information provided about methods used to assess the outcome (record "NR" as basis for answer).
Definitely High Risk of Bias	Direct evidence that the outcome assessment method is an insensitive instrument, OR the length of time after initial exposure differed by study group.

8. Were outcome data complete without attrition or exclusion from analysis?

Definitely Low Risk of Bias	Direct evidence that loss of animals was adequately addressed and reasons were documented when animals were removed from a study. Acceptable handling of attrition includes very little missing outcome data; reasons for missing animals unlikely to be related to treatment, missing outcomes does not impact the effect estimate.
Probably Low Risk of Bias	Indirect evidence that loss of animals was adequately addressed and reasons were documented when animals were removed from a study, OR it is deemed that the proportion lost would not appreciably bias results. This would include reports of no statistical differences in characteristics of animals removed from the study from those remaining in the study.
Probably High Risk of Bias or (NR)	Indirect evidence that loss of animals was unacceptably large and not adequately addressed, OR there is insufficient information provided about loss of animals (record "NR" as basis for answer).
Definitely High Risk of Bias	Direct evidence that loss of animals was unacceptably large and not adequately addressed.

9. Were all measured outcomes reported?

Definitely Low Risk of Bias	Direct evidence that all of the study's measured outcomes outlined in the protocol, methods, abstract, and/or introduction (that are relevant for the evaluation) have been reported.
Probably Low Risk of Bias	Indirect evidence that all of the study's measured outcomes outlined in the protocol, methods, abstract, and/or introduction (that are relevant for the evaluation) have been reported, OR analyses that had not been planned in advance (i.e., retrospective unplanned subgroup analyses) are clearly indicated as such and deemed that unplanned analyses were appropriate and selective reporting would not appreciably bias results (e.g., appropriate analyses of an unexpected effect). This would include outcomes reported with insufficient detail such as only reporting that results were statistically significant (or not).
Probably High Risk of Bias or (NR)	Indirect evidence that all of the study's measured outcomes outlined in the protocol, methods, abstract, and/or introduction (that are relevant for the evaluation) have not been reported, OR and there is indirect evidence that unplanned analyses were included that may appreciably bias results, OR there is insufficient information provided about selective outcome reporting (record "NR" as answer basis).
Definitely High Risk of Bias	Direct evidence that all of the study's measured outcomes outlined in the protocol, methods, abstract, and/or introduction (that are relevant for the evaluation) have not been reported.

Data Analysis and Evidence Synthesis

- The review team will qualitatively synthesize the body of evidence for each outcome and, where appropriate, a meta-analysis will be performed. If a meta-analysis is performed, summaries of main characteristics for each included study will be compiled and reviewed by two team members to determine comparability between studies, to identify data transformations necessary to ensure comparability, and to determine whether heterogeneity is a concern. The main characteristics considered across all eligible studies include the following:
    - Experimental design
    - Dose levels, frequency of treatment, timing, duration
    - Health outcome(s) reported
    - Type of data (e.g., continuous or dichotomous), statistics presented in paper, access to raw data
    - Variation in degree of risk of bias at individual study level
    - The review team expects to require input from subject-matter experts to help assess the heterogeneity of the studies. If a meta-analysis is conducted, a random effects model will be used for the analysis. Heterogeneity will be assessed using the I-squared statistic. Interpretation of I-squared will be based on the Cochrane Handbook: 0% to 40% (might not be important); 30% to 60% (may represent moderate heterogeneity); 50% to 90% (may represent substantial heterogeneity); and 75% to 100% (considerable heterogeneity). Additionally, as described in the Cochrane Handbook, for the last three categories, the importance of the I-squared will be interpreted considering not only the magnitude of effects but also the strength of the evidence (90% two-tailed confidence interval).
    - In the event that these proposed methods for data analysis are altered to tailor to the evidence base from included studies, the protocol will be amended accordingly, and the reasons for change will be justified in the documentation.

REFERENCES

Cottam N, Dodman MH. Comparison of the effectiveness of a purported anti-static cape (the Storm Defender®) vs. a placebo cape in the treatment of canine thunderstorm phobia as assessed by owners' reports. Appl Anim Behav Sci. 2009 119:78-84.

Cottam N, Dodman NH, Ha J. The effectiveness of the Anxiety Wrap® in the treatment of canine thunderstorm phobia: an open-label trial. J Vet Behav Clin Appl Res. 2013 8:154-161.

Diego MA, Field T. Moderate pressure massage elicits a parasympathetic nervous system response. Int. J. Neurosci. 2009 119: 630-638.

Edelson SM, Edelson MG, Kerr DC, Grandin T. Behavioral and physiological effects of deep pressure on children with autism: a pilot study evaluating the efficacy of Grandin's Hug Machine. Am J Occup Ther. 1999 Mar-Apr;53(2):145-52.

Fish RE, Foster ML, Gruen ME, Sherman BL, Dorman DC. Effect of wearing a telemetry jacket on behavioral and physiologic parameters of dogs in the open-field test. J Am Assoc Lab Anim Sci. 2017 56:382-389.

Higgins, J., and S. Green, eds. Cochrane Handbook for Systematic Reviews of Interventions, Version 6.4. The Cochrane Collaboration. Available online: http://handbook.cochrane.org (accessed on 26 November 2024).

Hooijmans, C.R., M.M. Rovers, R.B. de Vries, M. Leenars, M. Ritskes-Hoitinga, and M.W. Langendam. 2014. SYRCLE's risk of bias tool for animal studies. BMC Med. Res. Method. 14:43.

Koustas, E., J. Lam, P. Sutton, P.I. Johnson, D.S. Atchley, S. Sen, K.A. Robinson, D.A. Axelrad, and T.J. Woodruff. 2104. The Navigation Guide—evidence-based medicine meets environmental health: Systematic review of nonhuman evidence for PFOA effects on fetal growth. Environ. Health Perspect. 122(10):1015-1027.

NTP (National Toxicology Program). 2019. Handbook for Conducting a Literature-Based Health Assessment Using OHAT Approach for Systematic Review and Evidence Integration. Office of Health Assessment and Translation, Division, National Toxicology Program, National Institute of Environmental Health Sciences. Available at: https://ntp.niehs.nih.gov/sites/default/files/ntp/ohat/pubs/handbookmarch2019_508.pdf.

Pekkin AM, Hänninen L, Tiira K, Koskela A, Pöytäkangas M, Lohi H, Valros A. The effect of a pressure vest on the behaviour, salivary cortisol and urine oxytocin of noise phobic dogs in a controlled test. Appl Anim Behav Sci 2016 185: 86-94.

Williams NG, Borchelt PL. Full body restraint and rapid stimulus exposure as a treatment for dogs with defensive aggressive behavior: three case studies. Int J Comp Psychol. 2003 16: 226-236.

PROTOCOL AMENDMENTS

1. Addition of Suzie Schoolfield as a member of the review team

Table S1. List of studies that were excluded based on a review of the full text. The reason for exclusion is also provided.

Study	Reason for exclusion
Benito M, Lozano D, Miró F. Clinical evaluation of exercise-induced physiological changes in military working dogs (MWDs) resulting from the use or non-use of cooling vests during Training in moderately hot. environments. Animals (Basel). 2022 Sep 8;12(18):2347.	Wrong intervention – the study evaluated the use of cooling vests or pads rather than a compression wrap in dogs undergoing intensive work in a hot environment.
Chui RW, Fosdick A, Conner R, Jiang J, Bruenner BA, Vargas HM. Assessment of two external telemetry systems (PhysioJacket and JET) in beagle dogs with telemetry implants. J Pharmacol Toxicol Methods. 2009 Jul-Aug;60(1):58-68.	Wrong outcomes. This study compared heart rate and ECG endpoints following oral administration of either water or a therapeutic agent. Data obtained from each jacket system was compared with implant-derived data in the same animal. No external stressor was applied.
Riemer S. Effectiveness of treatments for firework fears in dogs.	Wrong study design. Review with no original data.
